# Young Adults’ Interactions With Food and Nutrition Content on Social Media and Implications for Intervention Design: Semistructured Interview Study

**DOI:** 10.2196/89344

**Published:** 2026-04-07

**Authors:** Hao Tang, Amy L Ahern, Marie Spreckley, Andrea D Smith

**Affiliations:** 1 Institute of Metabolic Science Epidemiology University of Cambridge Cambridge, England United Kingdom

**Keywords:** social media, young adults, qualitative, eating behavior, digital intervention, thematic analysis

## Abstract

**Background:**

Young adults increasingly rely on social media for nutrition information. However, little is known about (1) which types of eating-related content they actively engage with and why, and (2) how they interpret, evaluate, and incorporate this content into their everyday food choices and health behaviors.

**Objective:**

This qualitative study explored how UK young adults (aged 18-25 years) interact with food and nutrition content across social media platforms to inform the design of future social media interventions.

**Methods:**

Semistructured online interviews, guided by the Capability, Opportunity, Motivation–Behavior (COM-B) model, were conducted with 25 active social media users (18/25, 72% women, mean age 22.2, SD 1.9 years, ethnically diverse) in the United Kingdom between August and October 2024. The study design was informed by patient and public involvement to ensure relevance and acceptability. Data were analyzed using reflexive thematic analysis. To guide intervention development, key findings (coded as barriers and facilitators) were systematically mapped to the Theoretical Domains Framework, and the COM-B. Ethics approval was obtained from the University of Cambridge (24.368).

**Results:**

Five key themes were identified: (1) evolving engagement patterns (passive scrolling to active interaction and mixed feelings on algorithmic control), (2) conflicted information seeking (frustration with contradictory advice, varied strategies to assess credibility), (3) multifaceted behavioral impact (simultaneous positive impacts such as cooking inspiration and negative impacts such as restrictive eating triggers), (4) shifting goals (a movement from appearance-focused to health-centered goals; yet, vulnerability to body-image issues), and (5) intervention preferences (demand for credible professionals, customizable content, and privacy protection). Participants demonstrated a reactive learning process, developing “digital nutrition literacy” often after negative experiences. Social influences were identified as the most frequently cited domain (mapped to TDF [theoretical domains framework]/COM-B) shaping interactions with social media content.

**Conclusions:**

This study challenges assumptions of passive social media consumption, showing that young adults actively develop protective strategies yet remain vulnerable to misinformation. Digital interventions should leverage user agency and address diverse perceptions through customizable, credible content delivered with privacy and emotionally safe messaging. The COM-B and TDF mapping provide specific, evidence-based behavioral targets, particularly within the domain of Social Opportunity and Reflective Motivation, to guide the development of effective eHealth interventions.

**International Registered Report Identifier (IRRID):**

RR2-10.1136/bmjopen-2023-083465

## Introduction

Young adults (18-25 years) are immersed in a digital environment where social media platforms are a dominant presence. Social media use not only shapes their social interactions but also influences their eating behaviors and dietary choices [1-3]. As of 2025, there are more than 5.6 billion social media users, and nearly all (99%) internet users aged 16 years and older in the United Kingdom use platforms such as Instagram (Meta), YouTube (Google), and TikTok (ByteDance) [4,5]. These platforms are prominent and widely accessible sources of nutrition content, offering food-related material including influencer endorsements, recipe demonstrations, health challenges, and dieting trends [6,7].

The net influence of social media on dietary behaviors is contingent on multiple interacting factors, individual predispositions, and context. On the positive side, social media can serve as a space for peer support and positive dietary role modeling, enhancing access to practical nutrition information [7-9]. Health-promoting content was found to improve nutrition literacy and encourage the adoption of healthy behaviors, particularly when delivered by relatable peers [10,11]. Young adults also report that social media facilitates recipe discovery, meal-planning inspiration, and connection with online communities focused on wellness [12-14]. On the other hand, emerging research highlights that social media users are exposed to harmful dietary misinformation [15], including advice promoting restrictive dieting and elimination of food groups, and weight stigma, which may exacerbate body dissatisfaction and disordered eating behaviors [3,5,16].

Research also indicates that frequent social media use is associated with unhealthy eating behaviors, including increased intake of sugar-sweetened beverages (SSBs) and foods high in fat, sugar and salt (HFSS), lower fruit and vegetable consumption, and skipping meals [17,18]. Increasing exposure to online food content can both promote healthier eating and reinforce unhealthy food norms; yet, the balance of these effects remains unclear.

Despite growing interest in the relationship between social media and diet and eating behaviors, there remains a limited understanding of how young people actively engage with and interpret eating-related content online, and how such interactions can be leveraged to inform the development of effective digital interventions to support health promotion. Three critical gaps constrain efforts to address this public health challenge. First, food and nutrition content is among the most widely viewed topics on social media, with hashtags such as #WhatIEatInADay, #WeightLossFood, and #Recipes collectively generating billions of views across platforms. Yet most research relies on broad exposure measures and overlooks the nuanced ways in which young adults engage (eg, saving recipes, joining challenges, or curating creators on their feeds), underscoring the need for more behavior-specific investigation [19,20]. Second**,** while recent studies have linked social media use to both healthy and disordered eating behaviors, they offer limited insight into why these effects differ between individuals [3,21,22]. Previous reviews indicate that many rely on quantitative, cross-sectional designs using broad, decontextualized measures of social media use and often treat social media users as passive consumers without exploring the individualized nature of their engagement [3,23]. These limitations are particularly relevant during young adulthood, a formative period characterized by growing dietary autonomy, health awareness, and identity development [24-26]. Therefore, qualitative insights are needed to capture the mechanisms underlying how and why young adults engage with, interpret, and respond to nutrition content over time. Third, while behavior-change models, such as the Capability, Opportunity, Motivation–Behavior (COM-B) model, are widely recommended in health behavior research, their application in social media–based interventions remains limited [27-29]. One review noted that only 8 of 33 digital health interventions used theoretical frameworks [30].

To address these gaps, we conducted a theory-informed qualitative study, mapped to COM-B and TDF (theoretical domains framework), to explore how young adults interact with diet- and eating-related social media content and how these interactions shape behavior. Grounded in lived experience, the study identifies specific behavioral targets and opportunities for health promotion that extend beyond awareness-raising within today’s rapidly changing social media context.

## Methods

### Study Design

This qualitative study uses semistructured interviews to explore how young adults living in the United Kingdom engage with eating behavior-related content on social media. The interview guide was structured around the components of the COM-B model to provide a systematic framework for analyzing behavioral influences on dietary choices in digital environments [28]. The COREQ (Consolidated Criteria for Reporting Qualitative Research) checklist was used (Multimedia Appendix 1) [31].

### Patient and Public Involvement

Patient and public involvement (PPI) informed multiple aspects of this study, with PPI sessions conducted between March and May 2024. Four online sessions were held: 2 with members of the target population (UK-based social media users aged 18-25 years), 1 with a body-positivity social media influencer, and 1 with a staff member of a UK-based eating disorder charity. PPI feedback shaped the recruitment strategy. For example, participants recommended focusing on social media as the primary recruitment channel, prioritizing recruitment via Instagram posts and Facebook (Meta) groups and suggested additional relevant charities to contact. They also raised concerns about bot activity online, which led to the inclusion of a bot-filter question in the eligibility screening survey.

PPI members also contributed to refining the interview protocol, particularly for questions addressing potentially distressing or sensitive experiences. As a result, several questions were reworded for clarity and sensitivity. PPI contributors reviewed all participant-facing materials, including the interview guide, recruitment flyers, and the participant information sheet, and provided feedback on the clarity, tone, and cultural inclusivity of language used throughout. For example, a preliminary question was added asking participants to define what healthy eating meant to them personally before proceeding to questions about social media use, ensuring questions were grounded in participants’ own frameworks. Additionally, on the advice of PPI contributors, a sensitivity disclaimer was inserted before questions addressing disordered eating risk, informing participants that the following questions may be distressing and that they were free to skip or pause at any point.

### Participants and Recruitment

Participants were eligible if they were aged 18-25 years, lived in the United Kingdom, spoke English, and used social media (Instagram, TikTok, YouTube, etc) most days of the week. Recruitment was conducted from August to October 2024 through a multipronged approach, including posting on social media platforms (Instagram, Reddit, and Facebook groups) and posters in public locations. Interested individuals completed an online screening survey via REDCap (Research Electronic Data Capture; Vanderbilt University) to assess eligibility. From the pool of eligible respondents who provided authentic answers, participants were purposively selected to reflect diversity across gender, ethnicity, education level, and employment status. If individuals declined participation, replacements were drawn from the same demographic subgroup to maintain diversity.

The study sample size was informed by the concept of information power [32]. With the focused research aim, use of an established theoretical framework (the COM-B framework), and a demographically diverse sampling strategy, we aimed to recruit approximately 30 participants. Throughout data collection, we monitored the richness and relevance of interview material in relation to the study aim. We concluded the interviews when we felt that we had enough detailed interviews from diverse perspectives to provide rich insights into the research question, and had developed coherent analytic themes that answered the question, with depth and coverage across the components of the COM-B framework. These assessments were made through ongoing discussion between the lead researcher (HT) and the supervisory team (ALA, ADS, and MS), and data collection concluded at 25 interviews when these criteria were collectively satisfied.

### Data Collection

Semistructured interviews were conducted online via Microsoft Teams by a PhD researcher (HT) and were audio-recorded. Each interview lasted approximately 60 minutes. Field notes were made immediately after each interview to capture contextual information and initial reflections.

### Researcher Reflexivity Statement

The interviewer (HT) is a female PhD researcher in behavioral nutrition with training and prior experience in conducting qualitative interviews. Her background in nutrition and dietetics, and experience creating evidence-based content on social media, helped her understand participants’ experiences but also required awareness of potential assumptions about “healthy” content and online engagement. As someone with lived experience of navigating food and body image in the context of social media, HT was reflexively aware of potential shared experiences with participants and took care to remain open to perspectives that might differ from her own experiences. As a young adult and active social media user, HT shared generational proximity with participants, which may have facilitated rapport while also requiring reflexive awareness of shared assumptions. The power dynamics inherent in the researcher-participant relationship were acknowledged, particularly given the sensitive nature of topics such as body image and eating behaviors. HT kept brief reflexive notes after each interview and met regularly with supervisors (ALA and AS) to discuss any questions or uncertainties that arose during data collection.

### Interview Guide and Measures

The interview guide (Multimedia Appendix 2) covered topics including social media use patterns, experiences with healthy eating content, perceived influences on eating behaviors, platform preferences and engagement factors, and barriers and facilitators to healthy eating.

### Data Management

Audio recordings were transcribed and anonymized. All data were stored securely on the MRC (Medical Research Council) Epidemiology Unit Secure Research Drive (SRD), with access restricted to authorized members of the research team. Participants and their transcripts were assigned unique numerical IDs to ensure confidentiality.

### Data Analysis

#### Phase 1: Thematic Analysis

Data were analyzed using reflexive thematic analysis following Braun and Clarke’s six-phase approach [33], selected for its capacity to generate nuanced, participant-centered insights. HT led the analysis, using inductive coding of all transcripts in NVivo, whereby codes were generated in response to participants’ accounts without the imposition of a predetermined framework, and analytic themes were developed through an iterative process. To support analytic reflexivity and enrich interpretation, a subset of transcripts (5/25, 20%) was also coded by MS. These additional coding contributions informed collaborative discussions about alternative readings and identified patterns, contributing to the refinement of the thematic structure. The research team met regularly to discuss coding decisions and evolving themes, which supported reflexive engagement with the data and strengthened the coherence of the final analysis.

In this study, key concepts such as “healthy eating” and “credible content” were not defined a priori for participants during interviews. Instead, these were explored as open, participant-driven constructs, reflecting how individuals themselves interpreted and evaluated nutrition-related content on social media. For analytic consistency during subsequent COM-B and TDF mapping, references to “healthy eating” were considered in relation to broadly accepted UK public health dietary guidance [34], while remaining grounded in participants’ expressed meanings. “Credible content” was analytically defined as nutrition-related information that participants perceived as trustworthy, typically based on cues such as professional credentials, scientific references, tone, presentation style, or community validation.

#### Phase 2: Behavioral Framework Mapping

Identified determinants of young adults’ social media use were subsequently mapped to the TDF [35] and the COM-B model [28]. The COM-B model explains that for behavior change to occur, people must have the capability to change, the motivation to change and the opportunity to change. The TDF build on this by outlining 14 domains that capture the wider range of individual barriers and facilitators that may influence behavior. These domains systematically map to the COM-B model components (Multimedia Appendix 3). TDF and COM-B were selected because they support the translation of qualitative insights into behavioral domains and validated behavioral change mechanisms, which provide a strong foundation for future intervention development.

Each subtheme derived from the qualitative analysis was coded as either a barrier or a facilitator, or both where applicable, and subsequently categorized into the relevant TDF and COM-B domains. Influences could act in both directions depending on context and were coded as barrier/facilitator. Mapping barriers and facilitators to the TDF and COM-B allowed us to systematically identify which psychological, social, and contextual domains were most relevant to healthy eating within the social media context. Framework mapping was led by HT, with ADS providing an additional interpretative perspective. Interpretations were discussed iteratively to refine domain allocation and enhance conceptual clarity.

### Ethical Considerations

The study received ethical approval from the University of Cambridge School of Humanities and Social Sciences Research Ethics Committee (reference number 24.368). Prior to each interview, all participants provided informed consent electronically via a digital consent form. Participants were assigned unique numerical identifiers, and pseudonymized transcripts and personal data were stored separately and securely on the MRC Epidemiology Unit Secure Research Drive, accessible only to authorized members of the research team. All interview participants received a £25 (US $32) digital voucher as remuneration for their time, and all PPI contributors each received a £25 (US $32) digital voucher for their 1-hour session.

## Results

### Sample Characteristics

Of the 317 individuals who completed the initial online recruitment questionnaire, 277 met the eligibility criteria. A total of 133 responded to follow-up questions, and 30 individuals were scheduled for interviews. Among these, 27 participants joined the interview process. Two participants were excluded from the analytic sample: one whose responses did not sufficiently address the research questions concerning food and nutrition content on social media, and one who used racially offensive language that was inconsistent with the study’s ethical standards and respectful participation guidelines, resulting in a final analytic sample of 25 participants. Sufficient information power to answer our research question was achieved at 25 interviews. An overview of participant characteristics is provided in Table 1.

Participants had a mean age of 22.2 (SD 1.9) years and were predominantly women (18/25, 72%). The sample was ethnically diverse, comprising White (9/25, 36%), Asian/Asian British (8/25, 32%), Black/African/Caribbean/Black British (6/25, 24%), mixed/multiple ethnic groups (1/25, 4%), and other ethnic groups (1/25, 4%). Educational attainment varied, but most participants held a bachelor’s degree or equivalent (11/25, 44%) or had completed, or were enrolled in, postgraduate education (7/25, 28%).

In terms of occupation, 60% (15/25) were students, 16% (4/25) were employed full-time, 16% (4/25) were employed part-time, and 8% (2/25) were engaged in apprenticeships or traineeships.

All participants reported daily social media use, typically around 2-3 hours per day. Instagram (Meta) and TikTok (ByteDance) were the most frequently used platforms for accessing health- and nutrition-related content.

**Table 1:** Demographic characteristics of participants (N=25).^a^

Characteristics		Values, n (%)	
Age (years), mean (SD)		22.2 (1.9)	
**Gender**			
	Woman	18 (72)	
	Man	7(28)	
**Ethnicity**			
	Asian/Asian British	8 (32)	
	Black/African/Caribbean/Black British	6 (24)	
	White	9 (36)	
	Mixed/multiple ethnic groups	1 (4)	
	Other ethnic group	1 (4)	
**Occupational status**			
	Student	15 (60)	
	Employed full-time	4 (16)	
	Employed part-time	4 (16)	
	Apprenticeship or traineeship	2 (8)	
**Education level**			
	Postgraduate degree	7 (28)	
	Bachelor’s degree or equivalent	11 (44)	
	Post-secondary diploma or certificate	6 (24)	

^a^This table provides a detailed summary of demographic, educational, and occupational characteristics of the study sample. Continuous variables are presented as mean (SD), and categorical variables are presented as counts and percentages.

### Overview of Themes

**Figure 1:**
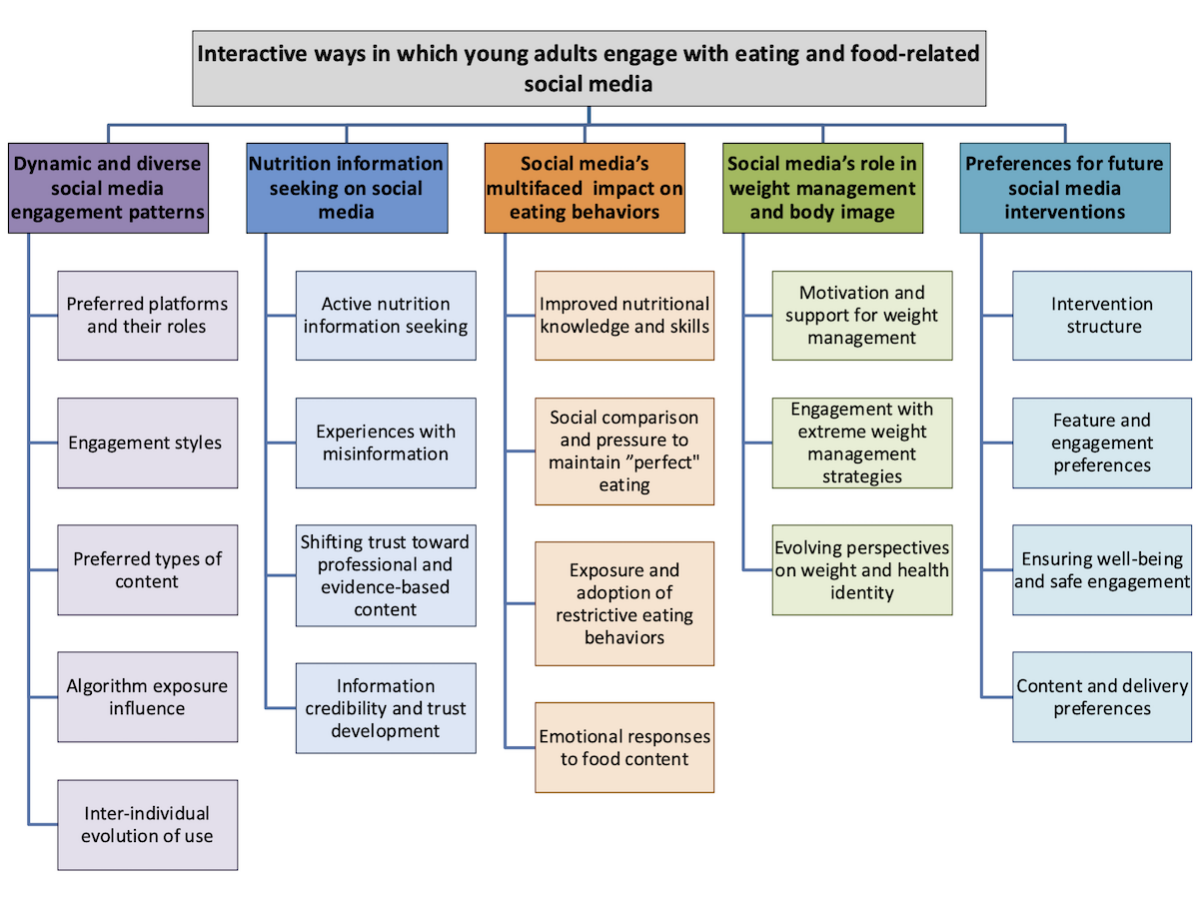
Thematic framework of young adults’ interactions with eating behaviour-related social media content.

Overall, five themes were identified, describing the diverse ways in which young adults engage with social media and how this influences their eating behaviors. Themes and subthemes are illustrated in Figure 1 and described in detail below.

### Theme 1: Evolving and Diverse Social Media Engagement Patterns

Participants described a range of ways in which they engaged with nutrition and dietary content on social media, emphasizing that their experiences were both highly personal and platform-specific. Engagement patterns were dynamic, evolving over time as participants developed preferences, adapted to changing algorithms, or sought to manage the emotional impact of content.

Platforms were associated with distinct benefits: Instagram was valued for community and peer support, YouTube for providing in-depth, educational material, and TikTok for engaging in short-form content. TikTok was also described by some as addictive or overwhelming. Engagement ranged from passive scrolling to active participation, such as commenting, voting in polls, or joining live streams.

Content appeal was strongly linked to visual engagement and relatability, such as the “What I eat in a day.” This video features creators, often celebrities or influencers, documenting their daily food and drink intake in a 24-hour time window. Participants perceived these videos as accessible and relatable because of their straightforward structure and perceived authenticity.

I watch a lot of food videos and get to know about different cultures. And it helped me realise the importance of adding vegetables in my diet…it helped me a lot. That was also where I learned to drink enough water instead of soft drinks. [HT prompted for example] I followed a marathon runner who’s vegan. I’m not attracted to veganism at all, but I liked her ideas of adding more vegetables in cooking. I remember watching her post about a chickpea sandwich, I know chickpeas are a good source of protein. It made me think it would be nice to eat less meat and try different food. She’s a well-known creator, so I trusted her, and I’ve actually made her recipes before.

Woman, 20 years old

However, not all experiences with social media content were positive. Some participants expressed frustration at the lack of control over what appeared on their feeds.

I watched a few fitness videos on TikTok last week and now my feed is just... lots of workout content and weight loss videos. It makes me feel kind of pressured to work out more, you know? …Sometimes I feel like I have no control over what I see; the algorithm decides for me.

Woman, 20 years old

Over time, some participants reported making deliberate adjustments to their social media use, such as unfollowing accounts, deleting apps, or reducing screen time, reflecting an active effort to manage their well-being.

I started to cut down my Instagram use because it made me feel uneasy about my own body.

Woman, 24 years old

### Theme 2: Nutrition Information Seeking on Social Media

Participants used social media as a primary source for quick nutrition information (eg, recipes and meal planning). However, utility was tempered by the prevalence of misinformation and conflicting dietary advice. Participants described adopting a number of strategies to verify information, such as checking credentials, cross-referencing sources, and reviewing comment sections, reflecting a developing “digital nutrition literacy.”

I often search on Xiaohongshu (also known internationally as RedNote) when I don’t know what to cook.

Man, 23 years old

Nevertheless, confusion remained high due to contradictory health claims online.

Too much conflicting information makes it hard to decide what’s right. Like, I saw posts saying that eating fruit is not healthy and doesn’t help digestion, and I saw other posts saying that drinking water immediately after eating is not healthy either. But also know from other sources that drinking water while eating is good for weight loss and fruit helps with digestion.

Woman, 24 years old

These were sometimes perceived as being driven by commercial interests rather than science.

You don’t know—some experts will say this is healthy, then a few years later, people change their minds. Like coffee—before it was bad, now they say it’s good for your heart. It’s confusing and feels like part of advertising.

Woman, 23 years old

Trust was influenced by both professional credentials and superficial cues such as the presenter’s appearance or the level of social validation (eg, positive comments) from the community.

Sometimes, when the influencer... looks healthy and positive, that helps [with credibility]. If they look really fit, I want to try their recommended snacks or diet…. I guess I think if they look that way, then what they’re recommending must work.

Woman, 25 years old

I look at the comments to see what other people have said. If most people say they got good results, then I’m more likely to trust what they (the creator/influencer) recommend.

Woman, 19 years old

### Theme 3: Social Media’s Multifaceted Impact on Eating Behaviors

The influence of social media was described as simultaneously positive and negative. Positive effects included skill development (eg, cooking techniques), improved knowledge, and inspiration for balanced meal choices.

I started cooking at home more after seeing easy and tasty recipes online.

Woman, 23 years old

Conversely, exposure to curated and unrealistic dietary and body ideals often led to social comparison, self-doubt, and the adoption of harmful behaviors, including restrictive eating, obsessive weighing, and calorie counting.

Seeing influencers made me feel inspired but sometimes made me question myself because they make diets look so easy.

Woman, 24 years old

When I just entered college, I gained a lot of weight and wanted to lose it. I found this influencer whose ‘what I eat in a day’ and weight loss tips videos I really enjoyed watching. She is a famous model. So I started trying the weight loss tips she recommended. After almost two months of skipping dinner due to posts promoting it as healthy, I developed eczema and felt horrible physically and mentally. That’s when I realised that these ‘model’ diets were very risky. So I stopped and unfollowed many accounts.

Woman, 25 years old

Reactions to specific content types were subjective. For example, mukbang videos (online broadcasts of individuals eating large quantities of food) elicited opposing responses: some participants felt they reduced cravings, while others found them uncomfortable or even disturbing.

### Theme 4: Social Media’s Role in Weight Management and Body Image

Social media played a shifting role in shaping participants’ body image concerns. Positive influences included motivation from success stories and support from health-focused communities.

I've been overweight since I was a child, and I started to feel really ashamed from my teenage years, like in middle school. I just couldn't get fit. I thought I was just lazy and born to be fat. Seeing success stories of people with obesity inspired me to start eating better.

Man, 25 years old

However, many participants recounted past experiences of body dissatisfaction exacerbated by unrealistic beauty standards promoted online. Over time, some reported deliberate progression from appearance-focused goals in their younger years to more balanced, health-centered approaches.

I think for me it used to affect it negatively… I saw models and pop stars… I wanted to be like that… I tried loads of diets like keto, detox, all kinds of things. Often I'd feel awful about myself, like, why can't I just be like them? There was also a lot of talk on social media saying these foods are bad for you, and because of that you have to punish yourself with workouts. It was a lot more unhealthy, and because of that, I think the impact was negative. Now I take from social media... making sure I’m eating enough. I think for me it used to affect it negatively, but I don’t think I realized that and that was when I was younger.

Woman, 25 years old

This shift demonstrates an evolving self-regulatory process aimed at mitigating the negative psychological effects of weight-centric content.

### Theme 5: Preferences for Future Social Media Interventions

Participants expressed a clear and diverse preference for future digital interventions, emphasizing that interventions should be short, visually engaging, and emotionally safe. Key preferences included short videos (30-60 seconds), involvement of professionals (eg, a qualified dietician) to build trust, interactive features (live Q&A sessions and journaling), and a privacy-protective environment (eg, anonymous chat).

*I think somethinganonymous and like no judgments could be useful. I think sharing eating or comment about eating can be sensitive*. [Woman, 24 years old]

Socializing within the programme can be really helpful to keep people tied because they can explore other personalities, or they can enjoy their time doing the programme.

Woman, 22 years old

Most participants preferred a time-limited intervention (2-4 weeks) with regular updates and structured, measurable goals to maintain motivation and prevent overwhelm.

### Mapping Identified Determinants of Eating and Food-Related Social Media Behaviors to the TDF

To inform the design of future interventions, key subthemes identified from the qualitative data were systematically mapped onto the TDF and subsequently COM-B. Each subtheme was evaluated and categorized as either a barrier or a facilitator to the use of social media interventions to promote healthy eating. A total of 106 barriers and facilitators were mapped across the dataset, with the largest proportion aligned with Motivation, followed by Opportunity and Capability.

Within the TDF, Social Influences (Opportunity) was the most frequently coded domain, reflecting the pervasive role of peers, influencers and community feedback in shaping young adults’ interactions with content, perceived credibility, and subsequent eating behaviors (peer influence leading to social comparison and community support for accountability). Barriers were concentrated in domains such as Environmental Context and Resources (Opportunity; eg, prevalence of misinformation and algorithm control) and Emotion (Motivation; eg, guilt and feeling triggered). Facilitators are frequently mapped to Knowledge (Capability) and Skills (Capability; eg, recipe demonstrations and critical evaluation skills) and Goals (Motivation; eg, shift toward sustainable health goals). These findings highlight that digital interventions need to target not only individual knowledge and skills but also the social and environmental context that dictates exposure and emotional response. Table 2 provides a summary of all barriers and facilitators alongside their theoretical domains. A comprehensive item-by-item mapping of all subthemes and barrier/facilitator pairs (n=106) is provided in Multimedia Appendix 4.

**Table 2:** Summary of key themes, barriers and facilitators to healthy eating when engaging with food and nutrition content on social media, mapped to Theoretical Domains Framework and Capability, Opportunity, Motivation–Behavior.

Themes^a^	Key findings	Primary barriers	Primary facilitators	TDF^b^ Domain (COM-B^c^)
Theme 1: Evolving engagement patterns	Participants used different platforms distinctly Algorithms shaped exposure positively and negatively	Algorithm-driven control Limited user autonomy over content exposure	Algorithm-driven tailoring. Platform affordances for engagement. Peer influence motivation.	Environmental Context & Resources (O); Beliefs about Capabilities (M)
Theme 2: Nutrition information seeking	High engagement but confusion from contradictory advice. Shift toward professional credibility	Misinformation prevalence. Difficulty evaluating contradictory claims	Critical-evaluation skill development. Preference for evidence-based sources. Professional credentials.	Environmental Context & Resources (O); Skills (C); Knowledge (C)
Theme 3: Multifaceted behavioral impact	Positive (skill development) and negative (social comparison and restrictive eating) impacts coexist	Unrealistic ideals Normalized restrictive eating Emotional triggers from comparison	Practical guidance. Knowledge access. Diverse content formats. Emotional support.	Social Influences (O) Emotion (M) Skills (C) Knowledge (C)
Theme 4: Weight management and body image	Evolving shift from appearance-focused to health-centered goals. Persistent vulnerability to ideals	Extreme dieting promotion. Unrealistic body ideals. Pressure to conform to thinness	Success stories. Community support. Skill development through shared techniques. Critical evaluation skills.	Social Influences (O) Beliefs about Consequences (M) Skills (C)
Theme 5: Intervention preferences	Strong demand for credible, customizable, emotionally safe content delivered in short formats	Algorithm opacity Content volume overwhelm	Short-form visuals (30-60 s). Professional delivery. Interactive features. Privacy protection. Time-limited structure.	Environmental Context and Resources (O) Knowledge (C) Emotion (M) Behavioral Regulation (C)

^a^This table summarizes the five key themes identified through reflexive thematic analysis, with primary barriers and facilitators identified within each theme and mapped to their corresponding Theoretical Domains Framework domains and Capability, Opportunity, Motivation–Behavior components. Subthemes within each theme have been condensed to show the most salient findings.

^b^TDF: Theoretical Domains Framework.

^c^COM-B: Capability, Opportunity, Motivation–Behavior.

## Discussion

### Principal Findings

This study highlights the complex and dynamic role of social media in shaping young adults’ eating behaviors, revealing a duality where platforms simultaneously facilitate healthy habits (eg, recipe discovery and positive motivation) and expose users to potentially harmful content (eg, misinformation, restrictive eating). Our findings, captured across five themes, detail the mechanisms of this influence. The COM-B/TDF mapping further identified that most barriers and facilitators fell under Motivation and Opportunity, underscoring that effective interventions must target Social Influences (a key Opportunity domain) and Reflective Motivation (eg, goals and beliefs about consequences), in addition to enhancing individual Capability. This highlights key leverage points for the design of future interventions.

### Comparison With Existing Literature

Consistent with previous literature, we found that social media can increase body image concerns and disordered eating risk, particularly among young women [3,36,37], while also supporting health-promoting behaviors and knowledge acquisition when used thoughtfully [38,39]. This study builds on these insights by emphasizing the importance young users place on credibility, a holistic approach, a nonjudgmental tone, and emotionally supportive environments.

This duality was clearly reflected in participants’ mixed experiences with evolving social media content, reflecting broader concerns about the overwhelming volume and variable quality of digital health information, which can make it difficult for users to distinguish trustworthy from misleading content [40,41]. This underscores the importance of equipping users with targeted digital health literacy skills and tailoring digital support programs to address the confusion arising from the volume and vagueness of online content [41-43].

This study advances current understanding in five ways. First, the study identified a dynamic behavioral pattern in how young adults engage with social media. Participants described a shift from appearance-related content in their earlier years (adolescence) to more health-focused, carefully evaluated use as they aged, especially after the transition to adulthood. This mirrors recent trends in UK youth, who have become more thoughtful and selective in their digital habits over time [23,44]. By capturing users’ reflective accounts and behavioral transitions, our study adds novel, person-centered insights with direct implications for tailoring digital interventions to users’ developmental stages rather than applying uniform approaches across all young adults.

Second, participants demonstrated sophisticated navigation strategies involving credential checking, community-feedback assessment, cross-referencing sources, and appearance-based judgments to evaluate nutrition information. These findings echo recent reviews calling for recognition of the varied and active ways young users engage with health information online, rather than characterizing them as passive consumers [45-47]. Liu and Zhang (2024) [48] observed similar deliberate credibility judgements in video-based content, while Kuutila et al (2023) [49] found that source expertise and prior belief alignment were stronger predictors of perceived credibility than evidence quality. While some critical evaluation skills were demonstrated (eg, credential checking and cross-referencing), our analysis also underscored the need for interventions that strengthen digital nutrition literacy by addressing the difficulty in evaluating contradictory claims.

Third, this study offers more nuanced insights into how young adults balance credibility and relatability when evaluating nutrition-related content online. While participants increasingly valued professional credentials and evidence-based sources, they remained highly engaged with content created by nonprofessional figures, such as influencers, models, and celebrities, including “what I eat in a day” videos or personal weight loss journeys, suggesting that digital health interventions may be more engaging when they intentionally combine both elements. Moreover, reactions to specific formats, such as weight-loss comparison and mukbang videos, varied widely. While some viewers described feeling comfort or inspiration, others felt guilt or discouragement. These findings echo recent studies [50-52], highlighting the heterogeneity in user responses and the need for personalized, context-sensitive intervention strategies.

Fourth, findings reveal a key tension in digital health engagement that challenges current intervention approaches. Participants appreciated algorithmic curation for efficiently surfacing relevant content but also expressed discomfort with its control over their exposure. Similarly, Milton et al [53] characterized the TikTok “For You” page as a “runaway train,” which is highly engaging but sometimes overwhelming and difficult to escape. Additionally, Savolainen and Ruckenstein [54] explored how users navigate algorithmic systems with varying degrees of autonomy, suggesting that while personalization can aid discovery, it often conflicts with self-directed control. These insights highlight a design challenge for digital health interventions, indicating that leveraging platform features for engagement should be balanced with mechanisms that preserve user autonomy and transparency.

Finally, this study highlights how people interact with different social media platforms in different ways. We found that in the context of dietary content, Instagram primarily fulfils community-building functions, YouTube mainly serves as a space for educational and in-depth content, and TikTok facilitates highly engaging, short-form delivery that can be overwhelming. These findings align with research suggesting that different platforms offer distinct affordances [55] and extend earlier qualitative work showing that young adults interact with healthy content differently across platforms [23]. This ecological perspective implies that effective digital health interventions should respect and leverage each platform’s unique characteristics rather than applying uniform approaches across all platforms.

### Insights for Social Media Intervention Design

This study offers several practical insights for the development of user-centered social media interventions to support healthy eating. A key finding was the high variability in content preferences, engagement styles, and emotional responses to dietary content, including mukbang videos and weight loss promotions. This highlights the need for customization options in future interventions. Such options may include adjustable content formats (eg, infographics and videos), frequency of posts, communication methods (eg, anonymous group chats, comments, and reposting), and types of health messaging (HAO Tang et al, unpublished data, August 2025)

Participants also expressed a preference for shorter content and multicomponent interventions, combining education with motivation and interaction. Realistic, relatable content delivered in emotionally safe tones (ie, nonjudgmental, supportive language that avoids triggering negative emotions or disordered eating behaviors) was consistently valued. Relatability was enhanced when content reflected the lived experiences of diverse individuals, rather than idealized or overly polished representations.

Our findings also identified a critical gap between information exposure and behavioral change. While social media served as a source of motivation and ideas, sustaining healthy habits remained challenging for many participants [56-58]. This highlights the importance of bridging the intention-behavior gap through established behavior-change techniques [59]. Our COM-B mapping results suggest that, while some young adults may possess adequate nutrition knowledge (psychological capability), more tailored strategies are needed to enhance Reflective Motivation and Social Opportunity for sustained behavior change. Specifically, interventions could incorporate goal-setting and self-monitoring (motivation/behavioral regulation) and use algorithm transparency tools to give users greater control over the content they are exposed to (physical opportunity). Social influence mechanisms may be targeted through leveraging credibility cues such as presenter credentials and community validation (eg, community moderation features and community comments).

The confusion participants experienced when navigating contradictory health messages points to a critical design requirement: interventions must equip users with frameworks or skills for evaluating conflicting advice rather than simply adding to the information volume [42,60]. This suggests incorporating features such as source-credibility checklists, decision-making tools that help users weigh competing claims, and educational modules that teach users to recognize common patterns in misleading nutrition content. Furthermore, many participants suggested that peer sharing, algorithmic visibility, and familiar platform features must be balanced with privacy considerations. The flexibility to choose levels of interaction (eg, passive viewing versus active commenting or posting) may help accommodate diverse comfort levels.

### Strengths and Limitations

This study’s strengths include the diverse sample achieved through purposive sampling, enhancing transferability within the UK young adult population. Comprehensive PPI throughout the research process strengthened the study’s cultural sensitivity and appropriateness, and applying a qualitative approach captured nuanced experiences otherwise not measurable using more quantitative approaches, essential for user-centered intervention design. The integration of COM-B and TDF provided both theoretical rigor and practical relevance, which paves a clear pathway for intervention development. This, coupled with the direct exploration of participant preferences for future interventions (Theme 5), offers practical guidance often missing from social media health research.

Some limitations should be noted. As participation was voluntary, the sample may be subject to selection bias, with perspectives potentially skewed toward those with a stronger interest or engagement in health-related topics or social media. Findings regarding specific platforms or content types need to be interpreted with caution, as the rapid evolution of social media technologies and user behaviors may limit their long-term applicability.

### Conclusion

This study provides new insights into how young adults in the United Kingdom interact with nutrition-related content on social media. Participants described a dynamic process of engagement shaped by evolving values, critical evaluation skills, and emotional responses. While social media can support healthy eating by offering accessible and motivational content, it also exposes users to conflicting messages and potentially harmful imagery and advice.

Findings highlight the importance of moving toward personalized digital health interventions that reflect users’ diverse needs, developmental stages, and evaluation capabilities. To increase the likelihood of achieving positive behavior change, interventions should combine credible, emotionally safe content with customizable features, privacy protection, professional oversight, and be grounded in established behavior change theory (COM-B/TDF). The tension between algorithmic curation and user autonomy suggests that successful interventions need to balance engagement optimization with user control and leverage multiplatform strategies that respect the unique and evolving features of different digital environments.

## Appendix

Multimedia Appendix 1 COREQ 32-item checklist.

Multimedia Appendix 2 Semi-structured interview guide used in the qualitative study.

Multimedia Appendix 3 Mapping of Theoretical Domains Framework (TDF) domains to COM-B components.

Multimedia Appendix 4 Detailed Item-Level Mapping of Barriers and Facilitators Healthy Eating When Engaging with Food and Nutrition Content on Social Media.

## Abbreviations

COM-B: Capability, Opportunity, Motivation and Behaviour model
COREQ: Consolidated Criteria for Reporting Qualitative Research
HFSS: high in fat, sugar, and salt
MRC: Medical Research Council
PPI: patient and public involvement
REDCap: Research Electronic Data Capture
SRD: Secure Research Drive
SSB: sugar-sweetened beverage
TDF: theoretical domains framework

## Data Availability

The terms of participant consent allow us to share deidentified qualitative data generated and analyzed during this study with researchers who are undertaking analyses related to the original objectives, with institutional approval. Requests should be directed to datasharing@mrc-epid.cam.ac.uk.
