# Supplementary material for: Young Adults’ Interactions With Food and Nutrition Content on Social Media and Implications for Intervention Design: Semistructured Interview Study
Source: J Med Internet Res. 2026 Apr 7;28:e89344. doi: 10.2196/89344 (PMC13100578; doi:10.2196/89344)
Supplement: Multimedia Appendix 2 [file jmir_v28i1e89344_app2.docx]

**Appendix 2: Interview protocol**

​​**Introduction** interviewer (HT):

Hi, thank you for taking the time to join this study. My name is Hao, a PhD researcher from the University of Cambridge, and I’ll be conducting this interview. It will take about 40-60 min. Today, I’d like to talk about your use of social media and how it's connected to your views on diet, your thoughts about food, and your eating habits. This insight is crucial as we aim to understand both the challenges and opportunities social media may present in promoting healthier eating habits. This is part of the SMILE study (**S**ocial **M**edia's **I**mpact on **L**ifestyle and **E**ating). If any question feels too personal or if you're uncomfortable at any point, please don't hesitate to let me know and we will stop the interview, or you can take a break before we resume (should you wish to).

And just so you know, there are no right or wrong answers here.

Should we lose connection, then I will attempt to re-contact you. And at the end of the interview, you will receive your £25 Amazon voucher via email as a thank you for your participation. Your personal experiences and views are what matter most to us. Ready to start?

Next, I will ask you a few questions about how social media shapes your eating attitudes and behaviours.

**Intro and Engagement with social media**

**Social media usage:** can you describe your daily use of social media? Which platforms do you prefer, and how much time do you spend on them?

What kind of content do you like to look at/follow?

- *Prompt:* News/Celebs/Lifestyle/Sports/ Art???

What about Health?

Linker section: In the next section, I will be asking about health-related social media.

**Capability**

1. What does "healthy eating" mean to you?
2. How has social media shaped your understanding of it? *Prompt: Can you give me an example?*
3. When you see information about eating on social media, do you find information it reliable, why?
   1. can you please share an example of when you found such information trustworthy or untrustworthy

**Opportunity**

1. How often do you see content about food or eating on social media? *Prompt: daily/weekly?*
2. What kind of content usually grabs your attention?
3. Can you describe a specific post that caught your eye recently? *Prompt: why did it stand out to you?*
4. Have you ever taken part in any social media challenges or campaigns about healthy eating (e.g. eating plans, go vegan..)?
   1. If yes, what was your experience like (prompt: Can you elaborate on what you feel enjoyed or found challenging about participating? *Did you cut out something from your diet?)*
   2. If not, what prevents you from joining (*Prompt:* *was it about intervention itself, the platform, or how you were feeling?*)

**Motivation**

1. What motivates you change your diet? (e.g., eat healthier, go vegan, reduce red meat, etc.)
2. Do you feel social media has influenced what you eat or/and how you think about food? (positively or negatively?).
   1. If yes : Can you share specific/recent examples of how it has affected your eating habits or choices, *Prompt: What, How, when, and why?*
3. Do you follow eating advice from social media? If yes, why, if no, why not?
   1. How do you feel about it? *(Prompt: challenging or straightforward for you to follow?)*
   2. Which platform(s) have more influence on your dietary decisions?
   3. Can you share a personal experience where you tried to follow eating advice from social media and how it went?
4. Moving forward, how interested are you in using and learning from social media about your eating habits? *Prompt: What motivates you more or less to use social media for this purpose?*
5. **(Sensitive question)** **I do want to talk about something sensitive about body image, please let me know if you want to stop. This is optional to answer:** Have you ever come across content on social media related to food, eating, or body image that made you feel uncomfortable, and triggered negative emotions about yourself?
6. As a result of this, do you feel like this has resulted in behaviours that have negatively affected your physical and mental health? *Prompt: : did you take any actions, like body checking, cutting out food groups, compulsive exercise, caloric restriction*
7. If you feel comfortable sharing, could you describe how you handled such situations? (e.g., seek professional help, talk with friends & family, follow body positivity influencers)
8. What kind of support or resources do you think would be helpful in those moments on **social media**? How do you feel about talk to an AI chat bot?

Note: Now for the final part, I just want to ask you to be creative about social media content.

**Designing a Social Media Program for Healthy Eating**

1. If you were designing social media interventions?
   1. What platform(s) do you prefer to use?
   2. What kind of social media content would you find most engaging and helpful for promoting healthy eating without harming your mental well-being? (e.g., podcast, videos with animation, videos with real people, words with pictures, voice)
2. What features or elements will be most helpful to keep you motivated and engaged? (to complete the program/stay part of the community)? (e.g., checklist, daily challenges, money incentives) If money is helpful, how much?
3. What length and frequency of the intervention would you prefer?
4. What format of information delivery would you prefer in a social media program for healthy eating? (e.g., real stories, practical tips, a combination of both)
5. Who would you like to communicate with in a social media program for healthy eating? (e.g., professionals, registered dietitians, researchers, peers, body positivity influencers workshops with Q&A Seed)
6. What forms of communication would you prefer in a social media program for healthy eating? (e.g., Chat bot, private messages, group chats, online communities)
7. Besides social media, what other forms of communication would you be comfortable with for receiving healthy eating support? (e.g., WhatsApp messages, email reminders)

**Closing**

HT: Thank you for sharing your valuable insights. Your input is incredibly important for our research and will help shape the design of future social media interventions. Before we conclude, is there anything else you'd like to share or any questions you have for me?

If you have any questions, please feel free to email Hao at [SMILE.study@mrc-epid.cam.ac.uk](mailto:SMILE.study@mrc-epid.cam.ac.uk).
