# Supplementary material for: Young Adults’ Interactions With Food and Nutrition Content on Social Media and Implications for Intervention Design: Semistructured Interview Study
Source: J Med Internet Res. 2026 Apr 7;28:e89344. doi: 10.2196/89344 (PMC13100578; doi:10.2196/89344)
Supplement: Multimedia Appendix 3 [file jmir_v28i1e89344_app3.docx]

**Appendix 3: TDF - COM-B mapping**

| TDF domain | Definition | COM-B component |
| --- | --- | --- |
| Knowledge | Awareness of the existence of something | Capability (C) |
| Skills | Ability or proficiency acquired through practice | Capability (C) |
| Social/Professional Role and Identity | Coherent set of behaviours and personal qualities in social or work settings | Motivation (M) |
| Beliefs about Capabilities | Acceptance of truth about an ability or facility that a person can put to constructive use | Motivation (M) |
| Optimism | Confidence that things will happen for the best | Motivation (M) |
| Beliefs about Consequences | Acceptance of truth about outcomes of behaviour | Motivation (M) |
| Reinforcement | Increasing the probability of a response by arranging contingencies | Motivation (M) |
| Intentions | Conscious decision to perform a behaviour | Motivation (M) |
| Goals | Mental representations of outcomes | Motivation (M) |
| Memory, Attention and Decision Processes | Ability to retain information and choose between alternatives | Capability (C) |
| Environmental Context and Resources | Circumstances that discourage or encourage behaviour | Opportunity (O) |
| Social Influences | Interpersonal processes that cause individuals to change thoughts or behaviours | Opportunity (O) |
| Emotion | Complex reaction pattern involving experiential, behavioural and physiological elements | Motivation (M) |
| Behavioural Regulation | Anything aimed at managing or changing objectively observed actions | Capability (C) |

C denotes Capability, O denotes Opportunity, M denotes Motivation. COM-B component labels are shown at the component level for clarity. Definitions based on Cane et al. (2012) and Atkins et al. (2017); COM-B components on Michie et al. (2011).
