# Supplementary material for: Young Adults’ Interactions With Food and Nutrition Content on Social Media and Implications for Intervention Design: Semistructured Interview Study
Source: J Med Internet Res. 2026 Apr 7;28:e89344. doi: 10.2196/89344 (PMC13100578; doi:10.2196/89344)
Supplement: Multimedia Appendix 4 [file jmir_v28i1e89344_app4.docx]

**Appendix 4 Detailed Item-Level Mapping of Barriers and Facilitators Healthy Eating When Engaging with Food and Nutrition Content on Social Media**

| **Subtheme** | **Barrier (B) /Facilitator (F)** | **TDF Domain (COM-B Domain)** |
| --- | --- | --- |
| **Theme 1: Evolving and Diverse Social Media Engagement Patterns** | | |
| **1.1 Preferred Platforms and Their Roles:**  Participants used different platforms for nutrition content in distinct ways. | B: Algorithm-driven content reinforced misinformation  F: Algorithm-driven tailoring increased engagement | - Environmental context and resources (O) |
|  | F: Already using social media frequently (existing habit) | - Behavioural regulation (C) - Environmental context and resources (O) |
| **1.2 Engagement Styles:**  Participants engaged in both passive and active ways, influenced by peers. | B: Interaction with other users on social media may bring peer influence and social comparison. | - Social influences (O) |
|  | F: Multiple ways for users to engage with social media | - Environmental context and resources (O) - Behavioural regulation (C) |
|  | F: Peer influence can be motivating | - Social influences (O) |
| **1.3 Preferred Types of Content:**  Short-form videos and real-life experiences were the most engaging content formats. Participants also appreciated educational posts, recipe videos, and fitness challenges. | F: Short-form videos are engaging | - Memory, attention and decision processes (C) |
|  | F: Real-life experiences increase relatability | - Social influences (O) |
|  | F: Recipe videos provide practical guidance | - Skills (C) |
|  | F: Educational posts build knowledge | - Knowledge (C) |
| **1.4 Algorithm Exposure Influence:**  Social media algorithms determined content exposure, shaping both positive and negative experiences. Participants felt limited control over what they saw. | B/F: Algorithms can determine what people are exposed to on social media | - Environmental context and resources (O) |
|  | B: Feeling limited control over what is seen | - Beliefs about capabilities (M) |
| **1.5 Inter-individual Evolution of Use:**  Participants’ engagement shifted over time from restrictive diet trends towards more balanced, sustainable approaches. | B/F: Users’ social-media habits evolve with age and environment | - Behavioural regulation (C) |
|  | B: Unfollowing influencers due to body-image concerns | - Social/professional role and identity (M) |
| **Theme 2: Nutrition Information Seeking on social media** | | |
| **2.1 Active Nutrition Information Seeking:**  Participants sought out nutrition-related content, including meal-prep ideas, diet plans, and expert recommendations. | F: Proactive search for nutrition-related content | - Memory, attention and decision processes (C) - Knowledge (C) |
|  | F: Interest in learning more about nutrition | - Goals (M) - Knowledge (C) |
|  | F: Availability of diverse information sources | - Environmental context and resources (O) |
| **2.2 Experiences with Misinformation:**  Participants had difficulty identifying and evaluating nutrition misinformation, leading to confusion and negative outcomes. | B: Prevalence and prominence of nutrition misinformation | - Environmental context and resources (O) |
|  | B: Difficulty evaluating contradictory claims | - Skills (C) - Knowledge (C) |
|  | B: Negative consequences from following incorrect advice | - Beliefs about consequences (M) - Beliefs about capabilities (M) |
| **2.3 Shifting Trust Towards Professional and Evidence-Based Content:**  Participants shifted from influencer advice to professional, evidence-based sources, preferring dietitians and health experts over unreliable trends. | F: Growing preference for credentialled experts | - Social influences (O) - Knowledge (C) |
|  | F: Recognition of the importance of scientific backing | - Beliefs about consequences (M) |
|  | F: Development of critical-evaluation skills with age | - Skills (C) - Beliefs about capabilities (M) |
| **2.4 Information Credibility and Trust Development:**  Participants trusted expert-backed content more than influencer-driven posts, with trust shaped by education, experience, and cues such as qualifications, tone, verification, and community feedback. (comments) | F: Research citations increase credibility | - Knowledge (C) - Beliefs about consequences (M) |
|  | F: Social validation through comments | - Social influences (O) |
|  | B/F: Presenter appearance or tone affects trust | - Emotion (M) - Social influences (O) |
|  | B: Superficial credibility markers may mislead | - Beliefs about consequences (M) |
| **Theme 3: Social Media’s Multifaceted Impact on Eating Behaviours** | | |
| **3.1 Improved Nutritional Knowledge and Skills:**  Social media enhanced nutrition knowledge, promoted healthier habits and cooking skills, and provided condition-management tips. | F: Free access to diverse nutrition information | - Knowledge (C) - Beliefs about capabilities (M) - Environmental context and resources (O) |
|  | F: Practical dietary tips and cooking demonstrations | - Skills (C) - Knowledge (C) |
|  | F: Exposure to varied and new cuisines | - Beliefs about consequences (M) - Environmental context and resources (O) |
| **3.2 Social Comparison and Pressure to Maintain ‘Perfect’ Eating:**  Exposure to idealised food content led to unhealthy comparisons with influencers, creating pressure for “perfect” diets and guilt over imperfect meals. | B: Unrealistic food presentation creates unhealthy standards | - Social influences (O) - Emotion (M) |
|  | B: Pressure to follow strict, ‘clean’ diets | - Social influences (O) - Social/professional role and identity (M) |
|  | B: Guilt when meals do not match idealised content | - Emotion (M) |
| **3.3 Exposure and Adoption of Restrictive Eating Behaviours:**  Some participants developed risky behaviours from idealised content, including restrictive eating, obsessive calorie counting, and meal skipping. | B: Social influence normalising restrictive eating | - Social influences (O) |
|  | B: Promotion of extreme dieting practices | - Beliefs about consequences (M) - Emotion (M) |
|  | B: Encouragement of unhealthy calorie/food measurement habits | - Behavioural regulation (C) - Social influences (O) |
| **3.4 Emotional Responses to Food Content:**  Participants showed distinct reactions to content such as mukbang, diet vlogs, and food challenges. For example, mukbang videos comforted some viewers but discouraged others from overeating. | B/F: Emotional responses to content vary | - Emotion (M) - Beliefs about consequences (M) |
|  | F: Some content may satisfy cravings | - Emotion (M) |
|  | F: Negative examples may discourage unhealthy eating | - Beliefs about consequences (M) |
| **Theme 4: Social Media’s Role in Weight Management and Body Image** | | |
| **4.1 Motivation and Support for Weight Management:**  Social media motivated participants by showcasing success stories, fitness journeys, and supportive communities. These positive influences helped some maintain accountability towards their health goals. | F: Success stories provide inspiration | - Reinforcement (M) - Social influences (O) |
|  | F: Community support increases accountability | - Social influences (O) |
|  | F: Access to diverse approaches | - Environmental context and resources (O) |
|  | F: Reinforcement through others’ experiences | - Reinforcement (M) - Social influences (O) |
|  | F: Skill development through shared techniques | - Skills (C) - Social influences (O) |
| **4.2 Engagement with Extreme Weight Management Strategies:**  Social media motivated weight control but also reinforced unrealistic ideals, pressuring participants into yo-yo dieting, obsessive exercise, and disordered eating. | B: Promotion of unrealistic body ideals | - Beliefs about consequences (M) - Social influences (O) |
|  | B: Normalisation of extreme dieting | - Social influences (O) - Emotion (M) |
|  | B: Young adults are particularly vulnerable for disordered eating | - Beliefs about capabilities (M) - Emotion (M) |
|  | B: Pressure to conform to thinness ideals | - Social/professional role and identity (M) - Social influences (O) |
| **4.3 Evolving Perspectives on Weight and Health Identity.**  Participants shifted from appearance-focused goals to balanced, sustainable approaches after recognising negative impacts of weight-centric content. | F: Development of critical-evaluation skills with age | - Skills (C) - Beliefs about capabilities (M) |
|  | F: Shift towards sustainable approaches | - Beliefs about consequences (M) - Goals (M) |
|  | F: Recognition of negative impacts of extreme methods | - Knowledge (C) - Beliefs about consequences (M) |
| **Theme 5: Preferences for Future Social Media Interventions** | | |
| **5.1 Content and Delivery Preferences:**  Participants favoured short, visually engaging content (30–60 seconds), balancing real stories for relatability and animations for neutrality. Evidence-based and practical information delivered progressively was highly valued. | F: Short, visually engaging content increases accessibility | - Environmental context and resources (O) - Memory, attention and decision processes (C) |
|  | F: Balance of relatability and neutrality | - Social influences (O) - Emotion (M) |
|  | F: Evidence-based, practical information | - Knowledge (C) - Skills (C) |
|  | F: Progressive information delivery prevents overwhelm | - Behavioural regulation (C) - Memory, attention and decision processes (C) |
| **5.2 Feature and Engagement Preferences:**  Participants favoured Instagram, with added interest in YouTube and web resources. They valued flexibility, privacy, interactive tools (Q&A, journaling), and both peer and professional support. | F: Instagram preference with complementary platforms | - Environmental context and resources (O) |
|  | F: Flexible schedules | - Environmental context and resources (O) |
|  | F: Privacy-protected interactions | - Social influences (O) |
|  | F: Interactive components (live Q&A, journalling, reposting) | - Behavioural regulation (C) - Social influences (O) |
|  | F: Peer support and professional guidance | - Social influences (O) |
| **5.3 Ensuring Well-Being and Safe Engagement:**  Participants emphasised balanced content to avoid triggering disordered eating, privacy protections such as anonymous chats, and expert moderation with clear disclaimers. | F: Content promoting health without triggering | - Emotion (M) - Environmental context and resources (O) |
|  | F: Anonymous options reduce embarrassment | - Emotion (M) - Social influences (O) |
|  | F: Expert moderation and disclaimers ensure safety | - Social influences (O) - Knowledge (C) |
| **5.4 Intervention Structure:**  Participants preferred 2–4-week interventions with regular updates, multiple communication channels, and structured, measurable goals to maintain motivation. | F: Time-limited interventions (2–4 weeks) | - Goals (M) - Behavioural regulation (C) |
|  | F: Regular updates maintain engagement | - Reinforcement (M) - Environmental context and resources (O) |
|  | F: Multiple communication channels | - Environmental context and resources (O) |
|  | F: Structured goals with measurable progress | - Goals (M) - Beliefs about capabilities (M) |

Mukbang: a combination of the Korean words “muk-ja” (eating) and “bang-song” (broadcast), referring to online videos or live streams where individuals eat large quantities of food while interacting with viewers.
